# FAM83D associates with high tumor recurrence after liver transplantation involving expansion of CD44+ carcinoma stem cells

**DOI:** 10.18632/oncotarget.12715

**Published:** 2016-10-18

**Authors:** Binyi Lin, Tianchi Chen, Qijun Zhang, Xiaoxiao Lu, Zhiyun Zheng, Jun Ding, Jinfeng Liu, Zhe Yang, Lei Geng, Liming Wu, Lin Zhou, Shusen Zheng

**Affiliations:** ^1^ Division of Hepatobiliary and Pancreatic Surgery, Department of Surgery, The First Affiliated Hospital, Zhejiang University, Hangzhou 310003, China; ^2^ Key Laboratory of Combined Multi-Organ Transplantation, Ministry of Public Health, Hangzhou 310003, China; ^3^ Collaborative Innovation Center for Diagnosis and Treatment of Infectious Diseases, Hangzhou 310003, China

**Keywords:** FAM83D, carcinoma stem cells, hepatocellular carcinoma, liver transplantation, CD44

## Abstract

To investigate the potential oncogene promoting recurrence of hepatocellular carcinoma (HCC) following liver transplantation (LT), throughput RNA sequencing was performed in a subgroup of HCC patients. The up-regulated FAM83D in HCC tissues was found and further verified in 150 patients by real-time PCR and immunohistochemistry. FAM83D overexpression significantly correlated with high HCC recurrence rate following LT and poor HCC characteristics such as high AFP, poor differentiation. Of cancer stem cells (CSCs) markers, CD44 expression was effectively suppressed when FAM83D was knocked down by siRNA. Meanwhile, the siRNA transfected cells suppressed formation of sphere and ability of self-renew. In a xenograft tumorigenesis model, FAM83D knockdown apparently inhibited tumor growth and metastasis. Microarray assays revealed that FAM83D promotes CD44 expression via activating the MAPK, TGF-β and Hippo signaling pathways. Furthermore, CD44 knockdown presented reverse effect on above signaling pathways, which suggested that FAM83D was a key activator of loop between CD44 and above signaling pathways. In conclusion, FAM83D promotes HCC recurrence by promoting CD44 expression and CD44^+^ CSCs malignancy. FAM83D provides a novel therapeutic approach against HCC recurrence after LT.

## INTRODUCTION

Hepatocellular carcinoma (HCC) is the sixth most common malignancy worldwide and the third most frequent cause of cancer deaths [1, 2], accounting for more than 70% of the total liver cancer, and epidemiologic evidence indicates that HCC will still increase medical and economic burden during the next decades [3]. Despite recently significant advances in screening, diagnosis and treatment for HCC, the survival time of a large proportion of HCC patients is significant low and the 5-year rate remains a dismal 12%. In theory, LT could simultaneously remove the tumor and underlying cirrhosis and don't need to consider the level of liver function impairment, which is currently the most effective therapy for HCC. Unfortunately, post-LT HCC recurrence and metastasis greatly give rise to the unsatisfactory long-term survival following LT. Furthermore, the number of HCC patients on the waiting line for LT and out of LT criteria is much more than liver recipients. Although several target drugs are under development, only sorafenib presented a finite survival benefits for individuals with advanced HCC [4]. Therefore, it is essential to find a potentially novel therapeutic target and predicting biomarker of diagnosis, treatment response and recurrence for improving HCC prognosis.

FAM83D that belongs to family with sequence similarity 83 genes locates on 20q. Amplification of chromosome 20q is frequently presented in various cancers and correlated with poor prognosis due to the ability of promoting cancer progression and metastasis [5]. Expectedly, several prior studies suggested that FAM83D is overexpressed in a variety of cancers including HCC, metastatic lung adenocarcinomas and ovarian cancer as a growth promoting gene [6–8]. A proteomic analysis of the human spindle apparatus revealed that FAM83D localizes to spindle microtubules regulating distribution of sister chromatids [9]. Furthermore, FAM83D coordinates chromokinesin Kid locating in spindle and generating polar ejection and chromosome congression [10]. Deficiency of FAM83D courses malfunctioning of the spindle leading to chromosome missegregation and aneuploidy, a hallmark of cancer. FAM83 protein family only contain a domain (DUf1669) with weak homology to a phospholipase D, and the elevated FAM83 member expression in tumor increase phospholipase D activity resulting in hyperactivation of epidermal growth factor receptor (EGFR) [11]. Cytoplasmic FAM83D interact with CRAF and promote CRAF translocation leading to activation of MAPK signaling spathways [12]. However, FAM83D whether associates with HCC recurrence after LT remains unclear, and the mechanism of FAM83D promoting HCC progress need to be further clarified. In the study, we found that FAM83D overexpression correlated with up-regulated CD44 expression.

HCC tissues are maintained in a hierarchical organization of heterogeneous groups of cancer cells including rare cancer stem cells (CSCs) with the ability of self-renewal and differentiation [13]. The existence of CSCs were verified in the context of various solid cancers and considered as one mechanism accounting for tumor growth and metastatic activity [14], and chemo-resistance and recurrence [15, 16]. The circular CSCs and extra-hepatic disseminated CSCs are able to survive for many years in a dormant state benefiting from their high resistance ability, and result in tumor relapse and renewed aggression after LT. Several prior studies identified CD133, CD44, CD24, CD90, CD13 and EpCAM as potential candidate CSC markers in HCC [17–22]. CD44 is a member of the cartilage link protein family and an important receptor of hyaluronan (HA). Alternative splicing could insert up to ten variant exon products into between domains 5and 6 leading to several CD44 variants. The crosstalk of CD44s or CD44v with tumor environment amplify a variety of signals endowing CSCs with special properties, such as epithelial-mesenchymal transition (EMT) and high resistance to apoptosis and niche preparation [22].

In the present study, we evaluated the effect of up-regulated FAM83D on HCC initiation, progression and recurrence and verified that FAM83D promotes CD44 overexpression via activating MAPK, TGF-β and Hippo signaling pathways.

## RESULTS

### The pattern and significance of FAM83D expression in HCC

**Figure 1:**
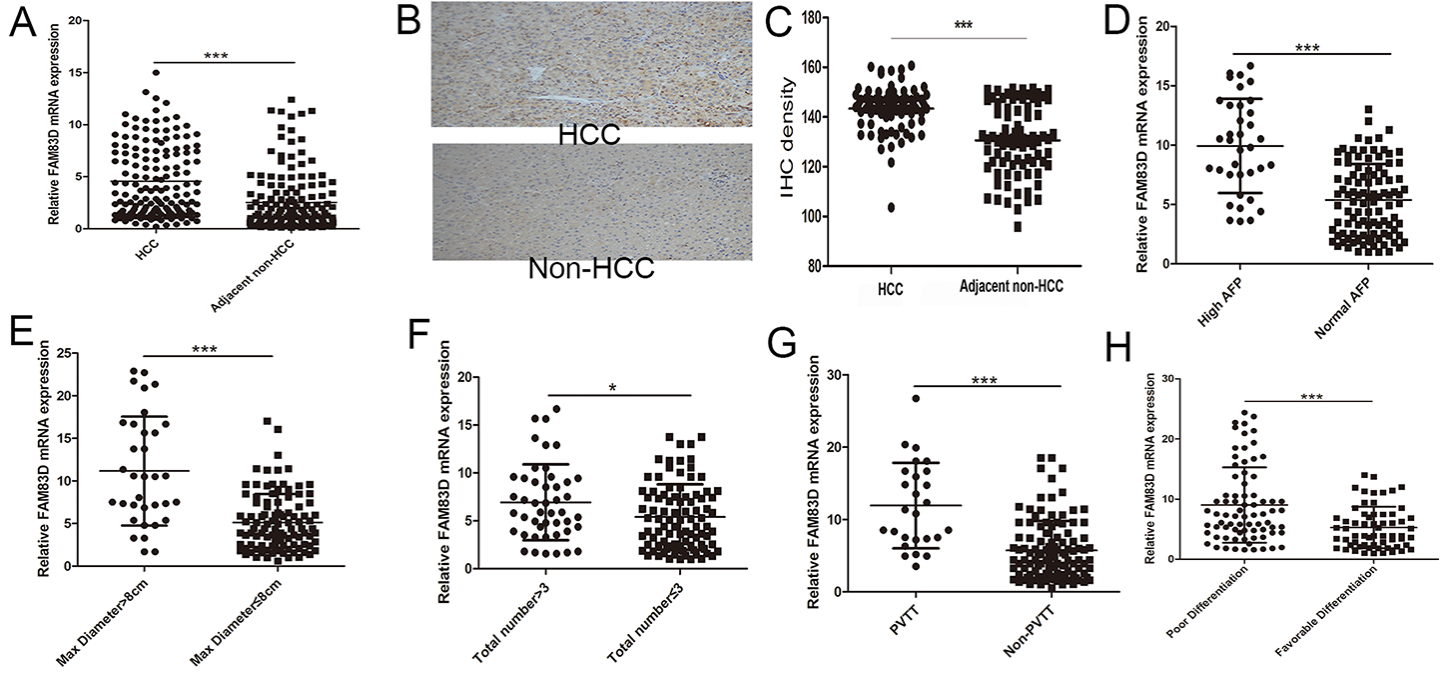
In 150 pair specimens, FAM83D was significantly overexpressed in HCC tissues comparing to adjacent non-HCC tissues **A. by RT-PCR**. This result was further confirmed by IHC **B, C.** Furthermore, further overexpression of FAM83D was found in tumors with higher AFP level **D.**, greater tumor diameter **E.**, more tumor number **F.**, portal vein tumor thrombus **G.** and poorer differentiation **H.**

**Figure 2:**
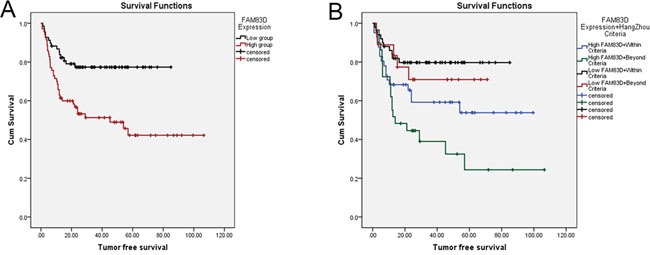
Kaplan-Meier's analysis disclosed that patients with high FAM83D expression developed more frequent HCC recurrence than low FAM83D expression **A.** In addition, HCC patients with low FAM83D expression maintained the advantage of tumor free survival no matter who was within or beyond LT Criteria **B.**

We profiled mRNA expression in liver samples from 10 HCC and corresponding non-HCC tissues (no pre-LT therapy) with RNA sequence and found that 321 up-regulated genes and 223 down-regulated genes (>2 folds, P<0.01). By pathway analysis, we defined the MAPK pathways is the most important in HCC initiation and development (Figure 1 in supplementary file). Among the 544 genes, FAM83D was increased in 8/10 HCC tissues compared with adjacent liver tissues. Additionally, we expansively detected the FAM83D expression in 150 pair specimens with real-time PCR and immunohistochemistry (IHC), and verified FAM83D was apparently up-regulated in HCC tissues (Figure 1A-1C). Moreover, overexpression of FAM83D in HCC was significantly correlated with poor tumor characteristics, i.e. poor differentiation, portal vein tumor thrombus (PVTT), tumor number, and greatest tumor diameter (Figure 1D-1H). Subsequently, Kaplan-Meier's analysis disclosed that patients with higher FAM83D developed more frequent HCC recurrence (Figure 2A) and presented poorer TFS outcomes after LT. The post-LT 5 years tumor-free survival rate was 75.4% in FAM83D-low group and 57.1% in the FAM83D-high group respectively. Besides, FAM83D-low patients showed better TFS no matter who was within or beyond LT Criteria of our center (Figure 2B). Cox's proportional hazards regression analysis revealed that high FAM83D expression was an independent predictor of prognosis of LT for HCC (TFS: hazard ratio [HR] 2.30; 95% confidence interval [CI] 1.15-4.60; P 0.019; Table 1).

**Table 1:** Predictors of HCC recurrence following LT

	Univariate analyses		Cox proportional hazards regression	
Variables	P value	HR(95%CI)	P value	HR(95%CI)0.017
Number	0.017	1.980(1.131-3.465)	0.625	1.163(0.634-2.136)
Diameter>8cm	<0.001	3.31(1.903-5.711)	0.032	2.129(1.067-4.248)
AFP	0.003	2.299(1.233-3.963)	0.667	0.861(0.434-1.706)
PVTT	<0.001	3.676(2.118-6.378)	0.010	2.265(1.213-4.23)
Differentiation	0.003	2.557(1.385-4.720)	0.316	1.482(0.687-3.19)
FAM83D	0.001	2.773(1.524-5.047)	0.019	2.3(1.15-4.6)

Note, HR, risk ratio, 95% CI 95% confidence interval

### FAM83D drives CD44 overexpression in HCC

**Figure 3:**
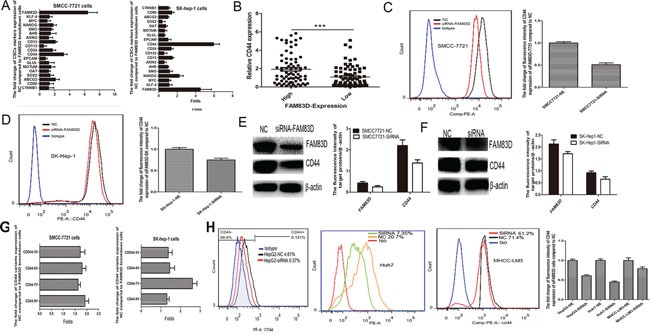
By RT-PCR, all of CSCs biomarkers, CD44 presented the greatest down-regulated folds when FAM83D expression was inhibited in SMMC7721 and SK-Hep-1 **A.** Furthermore, in the HCC patients with high FAM83D expression, the CD44 expression was apparently up-regulated **B.** FCA showed that enforced FAM83D knock-down significantly decreased the fluorescence intensity of CD44 expression **C, D.**, and the result was further verified by western blot **E, F.** Furthermore, FAM83D knockdown apparently reduced the all of CD44 variants expression **G.** In HepG2, Huh7 and MHCC-LM3 cells, FAM83D knockdown not only decreased the fluorescence intensity, but also could reduce the proportion of cells expressing CD44 **H.**

Given that CSCs played an important role in HCC progression and recurrence following LT and the high FAM83D expression in HCC positively correlated with AFP levels, we tested the relationship between FAM83D expression and CSCs biomarkers. A siRNA for FAM83D was transfected into SMMC-7721 and SK-Hep-1 cells with high FAM83D expression, and then the change of CSCs biomarkers expression were defined by RT-PCR. The results verified that FAM83D knockdown significantly decreased the CD44 expression (Figure 3A). Furthermore, in 150 HCC tissues, CD44 expression in the FAM83D high group was apparently higher than the FAM83D low group (Figure 3B). The lentivirus delivery of siRNA for FAM83D was used to achieve FAM83D knock-down HCC cell lines. Expression levels of FAM83D and CD44 were markedly down-regulated concurrently. SMMC-7721 and SK-hep-1 presented a 100% proportion of cells expressing CD44, FAM83D knockdown did not made a decrease in proportion of CD44 positive cells, but flow cytometric analysis (FCA) confirmed that enforced FAM83D knock-down lead to a significantly decrease in fluorescence intensity of CD44 expression (Figure 3C, 3D), and the result was further verified by western blotting (Figure 3E, 3F). In addition, the effect of FAM83D on CD44 variants expression was also detected, and confirming FAM83D knockdown apparently reduced the all of CD44 variants expression (Figure 3G). To further explore the effect of FAM83D on CD44 positive cells expansion, MHCC-LM3, Huh7 and HepG2 were transfected with siRNA for FAM83D, the results suggested that FAM83D knockdown not only decreased the fluorescence intensity but also could reduce the proportion of cells expressing CD44 (Figure 3H).

### FAM83D knockdown decreases stemness

**Figure 4:**
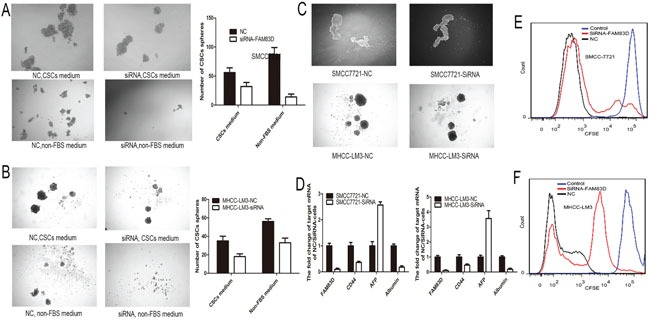
Suspension-cultured SMCC7721 and MHCC-LM3 cells with FAM83D knockdown presented lesser and smaller tumor spheroids than controls no matter in the non-FBS medium or CSCs medium **A-B.** Furthermore, the second formed spheroids were larger than first generation of spheroids, and FAM83D knockdown groups also presented smaller and lesser spheroids compared to NC groups **C.** Besides, decreased FAM83D expression increased the AFP expression and decreased the albumin expression in CSCs **D.** CFSE was used to trace cells and confirmed that FAM83D knockdown cells presented higher concentration of CFSE compared to NC after suspension-culture **E-F.**

Several previous published studies identified CD44 is a precise maker of CSC in HCC [22]. o clarify whether FAM83D knockdown inhibits HCC stemness, the spheroid formation assay in vitro that is a classic method to acquire CSCs [23] was performed. Suspension-cultured HCC cells with FAM83D knockdown had lesser and smaller tumor spheroids than controls (Figure 4A, 4B). Furthermore, the HCC cells spheroids were detached and re-suspended to form second spheroid in CSCs culture medium. The second formed spheroids were larger than first generation of spheroids, and FAM83D knockdown groups also presented smaller and lesser spheroids compared to NC groups (Figure 4C). Moreover, the AFP and albumin expression detection revealed that decreased FAM83D expression inhibited the differentiation of CSCs (Figure 4D). Besides, carboxyfluorescein diacetate, succinimidyl ester (CFSE) labeled cells verified FAM83D knockdown significantly reduced the self-renew after suspension-culture (Figure 4E, 4F). These results revealed that FAM83D knockdown effectively inhibited the stemness of CD44 positive CSCs.

### FAM83D knockdown inhibits tumorigenesis and metastasis of HCC in vitro and vivo

**Figure 5:**
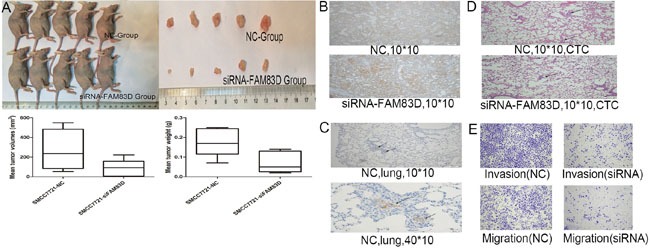
In vivo, FAM83D knockdown inhibited the tumorigenicity, and FAM83D-siRNA transfected SMMC-7721 cells presented an apparent decrease in tumor size and weight comparing the control cells **A.** Expectedly, FAM83D knockdown also reduced the CD44 expression in subcutaneously formed tumor tissues **B.** In NC group, at least one metastasis with high CD44 expression in lung was observed, but there was no pulmonary metastasis in the FAM83D knockdown group **C.** In the CTC model, the NC group presented more tumor colonies in lung than the FAM83D knockdown group **D.** Furthermore, in vitro, the transwell assay confirmed that knockdown of FAM83D would impact migration and invasion of SMCC7721 cells **E.**

The ability of tumorigenesis and tumor metastasis indicated the stemness of HCC cells with CD44 expression. We investigated the effect of FAM83D on tumor growth and metastasis in nude mice. FAM83D-siRNA-transfected SMMC-7721 cells and negative control cells were subcutaneously injected into nude mice (ten animals per group). Half of mice were sacrificed and the tumors were dissected after one month, remaining half of mice were sacrificed for observing the number of metastasis in lung after 1.5 month. Compared with the controls, FAM83D knockdown presented an apparent decrease in tumor size and weight (Figure 5A). The tumors were stained with CD44. Expectedly, tumors formed by FAM83D-siRNA-transfected SMMC-7721 presented decreased CD44 expression compared with controls (Figure 5B). Furthermore, at least one metastasis with high CD44 expression in lung was observed in the two mice of control group, but there was no pulmonary metastasis in the FAM83D knockdown group (Figure 5C). To further verify the effect of FAM83D on HCC cells colonizing in lung, we established a pulmonary metastasis tumor model using tail vein injection of HCC cells. Consistently, we found that FAM83D knockdown could inhibit tumor colonization in lung. In the control group, four of five mice presented multiple tumor colonies, but only two mice showed one to two tumor colonies in the FAM83D knockdown group (Figure 5D). Besides, the transwell assay confirmed that knockdown of FAM83D would impact migration and invasion of HCC cell lines in vitro (Figure 5E).

### FAM83D regulates multiple CSCs associated signaling pathways

**Figure 6:**
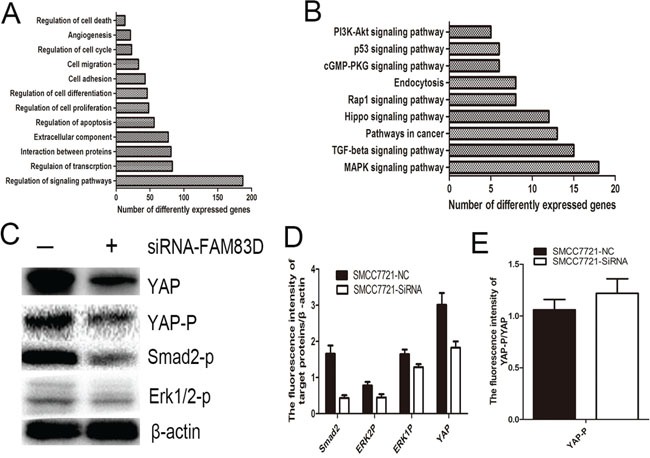
Gene expression microarray provided a total of 1565 differentially expressed genes The Kyoto Encyclopedia of Genes and Genomes (KEGG) analysis and gene ontology analysis revealed that FAM83D regulated multiple biological process such as cell migration, differentiation, adhesion **A.**, and multiple CSCs associated signaling pathways including TGF-β, MAPK, Hippo signaling pathways **B.** FAM83D knockdown reduced the levels of smad2 phosphorylation, ERK1/2 phosphorylation, decreased the YAP expression **C, D.** and promoted the proportion of phosphorylated YAP **E.**

Gene expression microarray was used to reveal the mechanism underlying FAM83D driving expansion of CD44 positive CSCs. SMMC-7721 cells transfected with siRNA-FAM83D resulted in a total of 1565 differentially expressed genes including 686 up-regulated genes and 879 down-regulated genes comparing with control cells. The data were further analyzed using an online database (http://david.abcc.ncifcrf.gov/) [24]. The gene ontology analysis disclosed that FAM83D knockdown made a deeply effect on multiple biological process including cell migration, differentiation, adhesion (Figure 6A). Furthermore, the Kyoto Encyclopedia of Genes and Genomes (KEGG) analysis indicated that FAM83D could regulate TGF-β, MAPK, Hippo signaling pathways (Figure 6B). By western blot, we verified that FAM83D knockdown reduced the levels of smad2 phosphorylation, ERK1/2 phosphorylation, decreased the YAP expression and promoted the proportion of phosphorylated YAP (Figure 6C-6E). The results suggested that FAM83D promoted CD44 expression via activating TGF-β, MAPK and Hippo signaling pathways.

### The inhibitors of MAPK, Hippo and TGF-beta signaling pathways suppress CD44 expression

**Figure 7:**
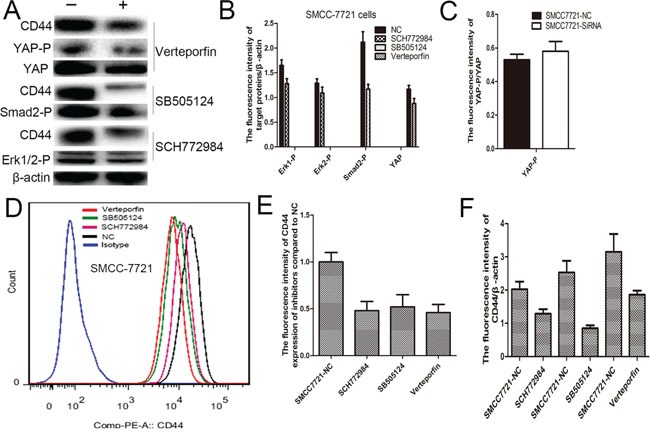
SCH772984, SB505124 and Verteporfin administration significantly inhibited the corresponding signaling pathways **A-C.** FCS confirmed that SCH772984, SB505124 and Verteporfin administration apparently reduced the CD44 expression **D, E.**, and western blot verified this result too **A, F.**

To further explore the effect of MAPK, Hippo and TGF-beta signaling pathways on CD44 expression, the SCH772984 (an inhibitor of ERK1/2 phosphorylation), SB505124 (an inhibitor of smad2/3 phosphorylation) and Verteporfin [25] (an inhibitor of YAP-TEAD interaction) were respectively used to inhibit corresponding signaling pathways (Figure 7A-7C), and results suggested that Verteporfin, SCH772984 and SB505124 could significantly suppressed fluorescence intensity of CD44 expression (Figure 7D-7E). These results were further verified by western blotting (Figure 7A, 7F).

### CD44 is a key upstream regulator of MAPK, Hippo and TGF-β signaling pathways

**Figure 8:**
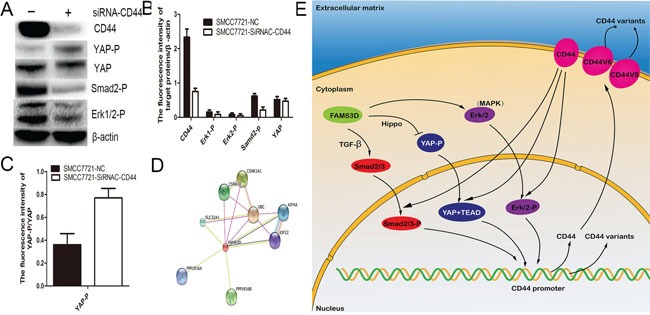
CD44 knockdown markedly reduced the level of ERK1/2 phosphorylation, Smad2 phosphorylation and YAP expression **A, B., and increased YAP phosphorylation C**. By an online database (http://string-db.org/), eight proteins including PPP1R16A, PPP1R16B, SLC32A1, CSNK1E, CSNK1A1, UBC, KIF4A, KIF22 were considered as potential protein interacting with FAM83D **D.** The map of molecular mechanism underlying FAM83D drives expansion of CD44 positive CSCs **E.**

Well known that CD44 is an importantly upstream regulator of multiple signaling pathways, the HA–CD44 or HA–CD44v interactions could promote activation of multiple kinases [22]. Therefore, a siRNA-CD44 transfection assay was used to investigate that the effect of CD44 knock-down on activation of MAPK, Hippo and TGF-beta signaling pathways. We found that CD44 knock-down significantly reduced the level of ERK1/2 phosphorylation, Smad2 phosphorylation and YAP expression, but increased YAP phosphorylation (Figure 8A-8C). These results suggested that CD44 is also a key upstream regulator of activating MAPK, TGF-beta and Hippo signaling pathways.

### The analysis of protein types interacting with FAM83D

An online database (http://string-db.org/) was used to investigate the protein types which potentially interact with FAM83D. Combing the data from a recent published article, we found potential nine proteins as follows, PPP1R16A, PPP1R16B, SLC32A1, CSNK1E, CSNK1A1, UBC, KIF4A, KIF22, CRAF (Figure 8D). Subsequently, these proteins were further analyzed by the online database (http://david.abcc.ncifcrf.gov/). The results of pathway analysis revealed that CSNK1E, CSNK1A1 involves in Wnt and Hedgehog signaling pathway, and CRAF is a promotor of MAPK pathways, but no proteins could regulates Hippo and TGF-β signaling pathways. These results potentially suggested that FAM83D activated MAPK signaling pathway via transferring location of CRAF in membrane leaded to decreased CD44 expression, and CD44 reduction inhibited TGF-β and Hippo signaling pathways (Figure 8E).

## DISCUSSION

Despite the great advances in surgical and immunosuppressors, the long-term survival of LT patients for HCC remains challenging due to a high incidence of HCC recurrence [26]. A better understanding of the molecular mechanisms underlying HCC metastasis will promote management and prognosis. To investigate the genes involving in the complicated metastasis process, we profiled the mRNA in HCC with RNA sequence. Following the pathway analysis, of the cancer related signaling pathways, the maximum genes located in the MAPK signaling pathway. Previously published data verified that FAM83 family bind CRAF to promote activation of MAPK signaling pathway, but the role and mechanism of FAM83 family in HCC was far clarified.

In this study, we confirmed that FAM83D was significantly overexpressed in HCC tissues compared with the adjacent non tumor tissues, and correlated with high HCC recurrence following LT. The TFS of the high FAM83D expression group was apparently poorer than the low FAM83D expression group, and the difference maintained in the HCC patients transplanted no matter beyond or within Milan criteria. These results suggested that FAM83D possibly is a precise indicator of improving selection of LT candidates. Furthermore, high FAM83D expression was verified correlating with tumor size, tumor number, PVTT and AFP levels. The AFP is produced by embryonic or infantile liver cells, which reminded that FAM83D potentially regulate CSCs functions.

Growing evidences demonstrated that CSCs possess the ability of self-renewal and give rise to anti-tumor drug resistance and tumor relapse, metastasis resulting in disease progression and motality [27, 28]. In despite of lacking simple, rigorous assays for defining the number of CSCs [29], measuring CSCs biomarkers potentially help clinicians to relist LT candidates. Llovet JM et al revealed that HCC patients without CSCs markers expression transplanted beyond Milan criteria achieved TFS rates similar as HCC patients transplanted within Milan criteria [30]. We investigated the role of FAM83D in CSCs. Of all CSCs biomarkers, CD44 expression was apparently down regulated following FAM83D knockdown. This result was further confirmed by FCS. FAM83D knockdown made a decrease in proportion of CD44 positive cells and a significantly decrease in fluorescence intensity of CD44 expression. In addition, we found that CD44 expression in HCC patients with high FAM83D expression was apparently higher than those with low FAM83D expression. In addition, FAM83D knockdown inhibited the ability of sphere formation, self-renew and differentiation. Besides, FAM83D knockdown HCC cells were injected into nude mice via tail vein or subcutaneously, and found that FAM83D knockdown inhibited the ability of tumorigenesis, pulmonary tumor colonization and tumor metastasis. These results suggested FAM83D is a promotor of CD44 expression and CD44^+^ CSCs malignancy.

CD44 attracted considerable interest for its abundant expression on CSCs [31], and overexpressed in local tumor tissues promoting tumor progression [22, 32, 33]. Recently, several articles revealed that targeting CD44 positive tumor cells with anti-CD44 monoclonal antiboby apparently inhibited proliferation and promoted apoptosis of sphere forming cells [34], and improved cells uptake of other anti-tumor chemicals [35], but the efficiency was influenced by CD44 isoform status [36]. Intriguingly, FAM83D not only inhibited the standard CD44 expression, but also down regulated the CD44 variants expression. Therefore, FAM83D potentially served as a novel target for improving the effect of anti-CD44 monoclonal antiboby therapy.

To explore the mechanism underlying FAM83D regulating the CD44 expression in HCC, a gene expression microarray assay was carried out. FAM83D knockdown inhibited MAPK, TGF-β and Hippo signaling pathway. Previously published data confirmed that these pathways were associated with CSCs phenotypes [37–39]. To our knowledge, only Arteaga, C. L et al revealed that DUSP4 loss promotes activation of MAPK pathways leading to increase CD44^+^CD24^−^cells population in basal-like breast cancer [37]. Currently, the role of MAPK, TGF-β and Hippo pathways in regulating CD44 expression in HCC was unclear. In the study, the SCH772984, SB505124 and Verteporfin were used to inhibit the corresponding signaling pathway, and we found that administrating Verteporfin, SCH772984 and SB505124 significantly inhibited the CD44 expression in HCC cell lines. These results firstly demonstrated the role of TGF-β and Hippo pathways in promoting CD44 expression.

Well-know that HA-CD44 and HA-CD44v interactions take a central role in multiple signaling pathways especially in receptor tyrosine kinase induced activation of pathways. A few articles revealed the interaction of CD44 and TGF-β, Hippo and MAPK signaling pathways. The interaction of CD44 and TGF-β pathways promoted breast tumor metastasis [40]. Xu Y et al demonstrated that CD44 attenuated phosphorylation level of the YAP leaded to activating Hippo pathway to enhancing efficacy of chemotherapy in glioblastoma multiforme [41]. P Zhao et al revealed that CD44 was necessary for MAPK pathways dependent lung adenocarcinoma proliferation [42]. To investigate the role of CD44 in regulating TGF-β, Hippo and MAPK signaling pathways in HCC, a siRNA-CD44 was transfected into HCC cell lines. We found that CD44 knockdown apparently activated Hippo pathway and inhibited MAPK and TGF-β pathways. These results confirmed that CD44 is also a key upstream regulator of activating MAPK, TGF-beta and Hippo signaling pathways.

Combining the data from recent articles and an online database, we found nine proteins potentially interacting with FAM83D. Among them, CSNK1E and CSNK1A1 activate Wnt and Hedgehog signaling pathways, CRAF promotes MAPK pathways, but no one stimulate Hippo and TGF-β signaling pathways. In the study, CD44 made a positive feedback with MAPK, Hippo and TGF-beta signaling pathways. Furthermore, FAM83D promoted the positive feedback leaded to CD44 overexpression and increased CSCs phenotype in HCC.

In summary, HCC patients with high FAM83D expression significantly correlate with the high recurrence rate of HCC following LT. The underlying mechanism involves FAM83D promoting expansion of CD44 positive CSCs via activating MAPK, Hippo and TGF-beta signaling pathways. FAM83D potentially provide a novel therapeutic approach against HCC recurrence after LT.

## MATERIALS AND METHODS

### Clinical specimen collection

The study was performed according to the ethical guidelines of the Declaration of Helsinki in 1975, and the experimental protocols were approved by the ethics committee of ZhangJiang University. Informed written consent was obtained from all LT patients. Samples from 150 HCC patients with LT at The First Affiliated Hospital, ZhangJiang University School of Medicine between 2006 and 2013 were collected for the study. Patients' data were followed and collected via hospital of information collection system of LT database.

### Cells culture

Seven human HCC cell lines (HepG2, Hep3B, Huh-7, SK-Hep-1, SMMC-7721, MHCC97H, MHCC97L and MHCC-LM3) and one immortalized normal liver cell line (LO_2_) were purchased from Cell Bank of Type Culture Collection of Chinese Academy of Sciences, Shanghai Institute of Cell Biology, Chinese Academy of Sciences and respectively cultivated according to the suppliers. HepG2, Hep3B, Huh-7, SK-Hep-1, MHCC97H, MHCC97L and MHCC-LM3 were cultured in the Dulbecco's modified Eagle medium (DMEM; Gibco-Invitrogen, Carlsbad, CA, USA) supplemented with 10% fetal bovine serum and 1% penicillin/streptomycin. SMMC-7721 and LO_2_ were cultured in 1640 complete medium supplemented with 10% fetal bovine serum and 1% penicillin/streptomycin. All cell lines were incubated in a humidified atmosphere of 5% CO_2_ at 37°C.

### Antibodies and chemicals

FAM83D antibody (Santa Cruz Biotechnology, CA, USA), Goat anti-Mouse HRP (ABCAM, Cambridge, MA, USA), β-actin antibody, CD44 antibody, Smad2/3 antibody, Smad2/3-P antibody, YAP/TAZ antibody, YAP-P antibody, ERK1/2 antibody and ERK1/2-P antibody, Goat anti-Rabbit HRP (Cell Signaling Technology, Beverly, MA, USA). Anti-CD44-PE, Anti-CD44-FITC (BD Pharmingen, NJ, USA). SCH772984, SB505124 and Verteporfin (Selleck Chemicals, TX, USA).

### Quantitative real time PCR (RT-PCR)

Total RNA from samples was extracted with a TRIzol RNA extraction kit (Invitrogen Co.). Reverse transcription was performed with a Rever Tre Ace-a-reverse transcription kit (Invitrogen Co.) to synthesize complementary DNA. The RT–PCR was performed with a Roche LightCycler using Takara SYBR Premix Extaq system. All preforming procedures were followed the instructions of manufactures. Primers were generated by Shanghai Sangon Biological Engineering Technology Services Co., Ltd. The nucleotide sequences of each primer presented in supplementary Table 1.

### Transfections of lentiviral vectors with FAM83D siRNA

To investigate the function of FAM83D on HCC, a FAM83D siRNA lentiviral vector (lenti-siRNA/FAM83D) was constructed (Shanghai GeneChem Co., Ltd., Shanghai, China), as well as a GFP-lentiviral vector with a scramble sequence (Scr-siRNA/GFP) as a negative control. Untreated SMMC-7721 cell and SK-Hep-1 cell at 5*105 were respectively plated into six-well plates for 24h. Monolayer cells per well with 20-30% confluency were transfected with lentiviral of lenti-siRNA/FAM83D or Scr-siRNA according to the manufacturer's instructions. To establish the stable cell lines, transfected cells were selected with 4 mg/ml of puromycin.

### Western blotting

Cells were collected and lysed in lysis buffer (Cell Signaling Technology, Beverly, MA, USA) at 4°C following the manufacturer's instructions. Western blotting analysis was performed according to the standard protocol. Briefly, protein concentrations were defined by a Bradford assay (Bio-Rad, Hercules, CA, USA) with a BCA Protein Assay Kit (Pierce, Rockford, IL, USA). Equal amounts of protein were separated by SDS-PAGE and then electrophoretically transferred to the 0.45um polyvinylidene fluoride (PVDF) membrane (Millipore, Bedford, MA, USA). The membrane was soaked and blocked with 5% nonfat milk in Tris-buffered saline supplied with 0.05% Tween-20 (TBST) at room temperature. After washing three times with TBST, membrane was incubated with the target primary antibodies (1:1000) overnight at 4°C, and experienced second washing and then incubated with the second corresponding antibodies (1:2000) at room temperature for 1 hour. The protein expression was visualized by SuperSignal West Pico Chemiluminescent Substrate (Pierce, Billerica, MA, USA).

### Immunohistochemistry

Immunohistochemical analysis of paired paraffin-embedded HCC tissues and adjacent normal tissues was carried out. Briefly, after experienced deparaffinating and rehydration, serial 4 μm sections then acquired heat-induced epitope retrieval. Next, 3% hydrogen peroxide was used to quench the endogenous peroxidase activity, and 5% FBS was used to block nonspecific binding. After blocking, the sections were incubated with primary monoclonal antibodies overnight at 4°C. The sections were incubated with secondary antibodies conjugated-HRP for 1h at room temperature and then visualized by 3,3'-diaminobenzidine, and followed by counterstaining with hematoxylin. Furthermore, phosphate-buffered saline replaced the primary antibody (PBS) to establish the negative control.

### Flow cytometry analysis

FAM83D-siRNA transfected cells or SCH772984, SB505124 and verteporfin treated cells were tested for the surface markers CD44 by incubating with anti-CD44-PE. Isotype controls were used to determine the background flurecscence. After washing second three times with PBS, CD44 expression was read in a 4-color FC500 flow cytometer (Beckman-Coulter, Miami, FL) for 1*10^4^ cellular events.

### Tumorsphere formation assay

To testing growth of primary tumorsphere, 1000 cells (Control cells, FAM83D-siRNA transfected cells) were seeded in 6-well plates with ultralow attachment (Corning, Lowell, MA, USA) and grown in serum-free 1640 medium or tumorsphere forming 1640 medium supplied with serum-free DMEM/F12 supplemented with human epidermal growth factor, insulin and B27 (Gibco-Life Technologies, Carlsbad, CA). The size and number of tumorsphere were analyzed in culture using an inverted microscope after 7-10 days.

### Tumor xenograft experiment

All the experiments procedures were performed in accordance with the guidelines for the National Institutes of Health guide for the care and use of laboratory animals. Firstly, 5*106 cells/mice were injected subcutaneously in the lateral flank of immunodeficient mice. Tumor volume (measured weekly with a caliper) was calculated using the equation: Tumor size = (Width^2^ *Length)/2. After 4 weeks, half of Mice were sacrificed and tumors were harvested for immunostaining after cell injection, and other half of mice were sacrificed and defined the number of lung metastasis after 6 weeks. Furthermore, for testing the ability of tumor colony in lung, 1*10^6^ cells/mice were injected into immunodeficient mice via tail vein. After 6 weeks, Mice were sacrificed and the numbers of tumor colonies in lung were analyzed by haematoxylin eosin staining.

### Cell migration and invasion assays

For the transwell migration assays, 3*10^4^ SMMC-7721 cells in serum-free medium were plated in the upper chamber with a non-coated membrane (24-well insert; 8-mm pore size; Millipore, Billerica, MA, USA). For invasion assays, 3*10^4^ SMMC-7721 cells in serum-free medium were placed in the upper chamber with a Matrigelcoated membrane. In both assays, the lower chamber were filled with 500ul culture medium supplied with 10% FBS. After incubation for 24 h, 48 h and 72h (migration and invasion assay), cells on the lower membrane were counted by fixed with 95% methanol and stained with 0.2% crystal violet.

### Statistical analysis

All of experiments were performed thrice and the data were presented as mean ± SD or frequency. A two-tailed Student's t test and Fisher's exact test were performed to analyze the potential association between FAM83D and clinicopathologic parameters, malignant phenotype. Tumor free survival (TFS) was calculated from the date of LT until HCC recurrence. Potential predictive factors were analyzed by Kaplan-Meier curves and the log-rank test. Preoperative factors with significance (p< 0.10) for TFS in the univariate analysis were further selected into the Cox proportional hazards model (backward selection likelihood function) to determine their independent effects. SPSS19.0 software was used for statistical analysis and P< 0.05 was considered as statistical significance.

## SUPPLEMENTARY MATERIALS FIGURE AND TABLE


