# Supplementary material for: FAM83D associates with high tumor recurrence after liver transplantation involving expansion of CD44+ carcinoma stem cells
Source: Oncotarget. 2016 Oct 18;7(47):77495–507. doi: 10.18632/oncotarget.12715 (PMC5363599; doi:10.18632/oncotarget.12715)
Supplement: Supplementary file 1 [file oncotarget-07-77495-s001.pdf]

FAM83D associates with high tumor recurrence after liver transplantation involving expansion of CD44^{+} carcinoma stem cells

SUPPLEMENTARY FIGURE AND TABLE

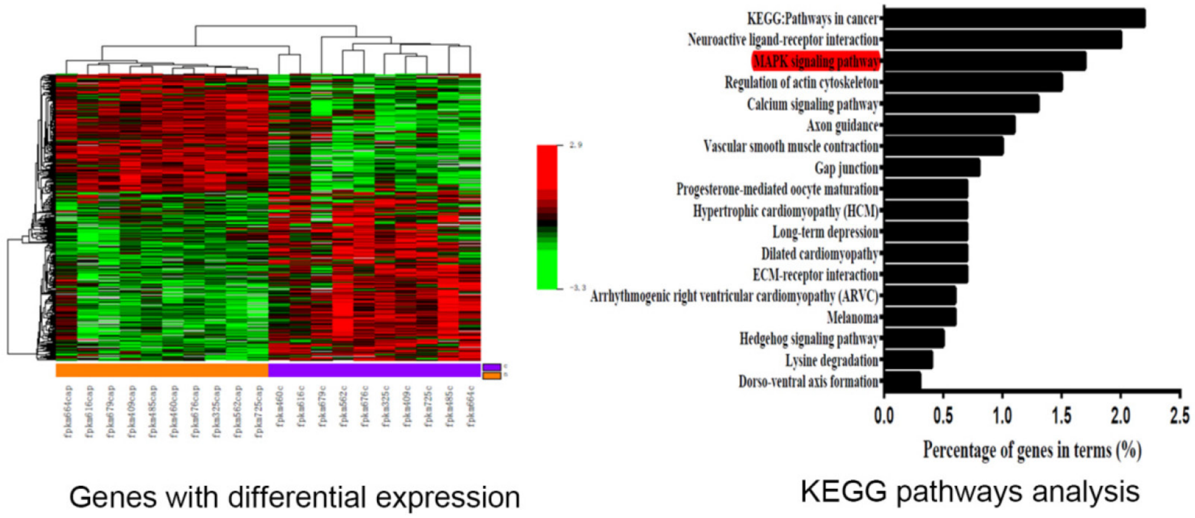

Supplementary Figure S1: The 10 pairs of HCC and corresponding non-HCC tissues (no pre-LT therapy) were treated with RNA sequence. The mRNA with differential expression were profiled, and found that 321 up-regulated genes and 223 down-regulated genes (>2 folds, P<0.01). By pathway analysis, we defined the MAPK pathways is the most important cancer related signaling pathways in HCC initiation and development. Among the 544 genes, FAM83D was increased in 8/10 HCC tissues compared with adjacent liver tissues.

Supplementary Table S1: The nucleotide sequences of each primer

Genes	Sequence
CD133 forward	CACTCTATACCAAAGCGTCAA
CD133 reverse	CACGATGCCACTTTCTCAC
CD90 forward	GACCCGTGAGACAAAGAAGCA
CD90 reverse	GAGGAGATGGGTGGGGAAT
CD44 forward	GTTCCTGGACTGATTTCTTC
CD44 reverse	ATTACTCTGCTGCGTTGTC
EPCAM forward	GTTGTTGCTGGAATTGTTGT
EPCAM reverse	CATCTCACCCATCTCCTTTAT
CD13 forward	GCCACCTCTACCATCATCAGC
CD13 reverse	TGTTTCCTCGTTGTCCTTCTTG
CD24 forward	GCTCCTACCCACGCAGATTT
CD24 reverse	CCTTGGTGGTGGCATTAGTT
Oct-4 forward	TATTCAGCCAAACGACCATCT
Oct-4 reverse	ACGAGGGTTTCTGCTTTGC
Nanog forward	CCGAAGAATAGCAATGGTG
Nanog reverse	CCTGGTGGTAGGAAGAGTAAA
Sox2 forward	ATGGGTTCGGTGGTCAAGTC
Sox2 reverse	GCTCTGGTAGTGCTGGGACAT
ABCG2 forward	ACTCAGTTTATCCGTGGTGTG
ABCG2 reverse	CCTGCTTAGACATCCTTTTCA
GPC3 forward	AAATACCAACTAACAGCACGAT
GPC3 reverse	ATTCACCCACAAACTCAAAA
CD44s-sense	AAG ACA TCT ACC CCA GCA AC
CD44v3-sense	ACG TCT TCA AAT ACC ATC TC
CD44v6-sense	CAG GCA ACT CCT AGT AGT AC
CD44v7-sense	CAG CCT CAG CTC ATA CCA GC
CD44v9-sense	CAG AGC TTC TCT ACA TCA CA
CD44s-antisense	CCA AGA TGA TCA GCC ATT CTG G
Homo-FAM83D (forward)	AGTTCCGAATCCTGTATGCC
Homo-FAM83D (reverse)	GCTCCTTGGACTGTGGTTT
